# Transcriptional changes of biochemical pathways in *Meloidogyne incognita* in response to non-fumigant nematicides

**DOI:** 10.1038/s41598-022-14091-3

**Published:** 2022-06-14

**Authors:** Catherine L. Wram, Cedar N. Hesse, Inga A. Zasada

**Affiliations:** 1 Department of Botany and Plant Pathology, Oregon State University, Corvallis, OR 97331 USA; 2 USDA-ARS Horticultural Crops Research Unit, Corvallis, OR 97330 USA

**Keywords:** Target identification, Toxicology, Agricultural genetics, Gene expression, Sequencing

## Abstract

*Meloidogyne incognita* is a destructive and economically important agricultural pest. Similar to other plant-parasitic nematodes, management of *M. incognita* relies heavily on chemical controls. As old, broad spectrum, and toxic nematicides leave the market, replacements have entered including fluensulfone, fluazaindolizine, and fluopyram that are plant-parasitic nematode specific in target and less toxic to applicators. However, there is limited research into their modes-of-action and other off-target cellular effects caused by these nematicides in plant-parasitic nematodes. This study aimed to broaden the knowledge about these new nematicides by examining the transcriptional changes in *M. incognita* second-stage juveniles (J2) after 24-h exposure to fluensulfone, fluazaindolizine, and fluopyram as well as oxamyl, an older non-fumigant nematicide. Total RNA was extracted and sequenced using Illumina HiSeq to investigate transcriptional changes in the citric acid cycle, the glyoxylate pathway, $\upbeta$-fatty acid oxidation pathway, oxidative phosphorylation, and acetylcholine neuron components. Observed transcriptional changes in *M. incognita* exposed to fluopyram and oxamyl corresponded to their respective modes-of-action. Potential targets for fluensulfone and fluazaindolizine were identified in the $\upbeta$-fatty acid oxidation pathway and 2-oxoglutarate dehydrogenase of the citric acid cycle, respectively. This study provides a foundation for understanding how potential nematicide resistance could develop, identifies cellular pathways as potential nematicide targets, and determines targets for confirming unknown modes-of-action.

## Introduction

Plant-parasitic nematodes are some of the most devastating agricultural pests^1^. The southern root-knot nematode, *Meloidogyne incognita,* is of particular importance due to its vast host range and widespread distribution^1–6^. *Meloidogyne incognita* is endemic in at least 72 countries, mostly in tropical and sub-tropical climates^6^. Chemical controls remain the predominate method for controlling plant-parasitic nematodes due to their high efficacy and challenges facing the development and use of other control methods^7^. Over the last 30 years however, the chemical control market has shifted focus from broad-spectrum soil fumigants with activity on weeds, fungi and nematodes, to non-fumigant nematicides with more focused targets^7,8^. This drive for compounds targeted exclusively at plant-parasitic nematodes is due to the adverse effects both on human health and the environment that soil-fumigants have posed. This phase out of potentially harmful chemical controls is not limited to soil fumigants, but includes nematicides registered over 50 years ago, like the carbamate oxamyl and the organophosphate fenamiphos^8^. These compounds act as acetylcholinesterase inhibitors and can have toxic effects on nematodes, insects, and humans^9^. Restrictions on the use of carbamates and organophosphates have been put into place to reflect the human and environmental toxicity of these compounds^8,10^.

To fill this gap in plant-parasitic nematode chemical control, several new nematicides have come to market since the late 2000s, including fluopyram, fluensulfone, and fluazaindolizine. These nematicides have reduced user warning labels and directly target plant-parasitic nematodes^8^. These three new nematicides share a trifluoro group in their chemical structure, but their physical properties, soil half-life, nematicide efficacy, and other properties are vastly different^8^. Fluopyram, a pyridinyl-ethyl benzamide, has some suppressive activity against plant-parasitic nematodes, but is mainly used in agriculture as a fungicide. In both fungi and nematodes, this compound functions as a succinate dehydrogenase inhibitor (SDHI)^11,12^. In in vitro studies fluopyram was nematistatic; *M. incognita* second-stage juveniles (J2) were able to recover mobility after a 24 h exposure and subsequent removal of the compound at doses < 6 ppm^13,14^. As a reflection of fluopyram’s weak in vitro effects on plant-parasitic nematodes, control was variable when applied in the field or greenhouse with little to no suppression to significant reductions in nematode reproduction^14–18^.

Fluensulfone is a member of the fluoroalkenyl thioether group and is considered a true nematicide^19^. In in vitro studies, *M. incognita* exposed to fluensulfone were unable to recover even after the compound was removed^14,20^. In field trials, fluensulfone effectively reduced root galling caused by *M. incognita* on cucumber, carrot, and lima bean and in some cases reduced *M. incognita* J2 population densities^15,16,18,21^. Fluensulfone has an undescribed mode-of-action. Both *Caenorhabditis elegans* and *M. incognita* responded in a comparable manor when exposed to similar doses of fluensulfone^19^. Additionally, *C. elegans* mutants resistant to organophosphates and carbamates were susceptible to fluensulfone, indicating cholinesterase inhibition is not the target for this compound^19^.

Fluazaindolizine is an imidazopyridine discovered in 2017^22^. Like fluensulfone and oxamyl, its effects on PPN are irreversible^23^. However, compared with other nematicides in this study, in in vitro assays fluazaindolizine was slower acting, with a 24-h EC_50_ (effective concentration that resulted in 50% of the population becoming inactive) approximately 2 × that of fluensulfone and oxamyl, and 200 × that of fluopyram^14^. Fluazaindolizine appears to have a limited scope of efficacy, with some species of plant-parasitic and free-living nematodes unaffected by exposure^23,24^. *Caenorhabditis elegans* and *Drosophila melanogaster* were not impacted by fluazaindolizine^22^. Lahm et al. used in vitro assays to determine if fluazaindolizine had a similar mode-of-action to current nematicides. Acetylcholinesterase, mitochondrial electron transport chain, nicotinic acetylcholine receptors, or glutamate-gated chloride channels were unaffected by this compound. Currently the mode-of-action of fluazaindolizine is still unknown.

As nematicides on the market for managing plant-parasitic nematodes are reduced with limited replacements, there are concerns that complications with pesticides in other systems could begin to occur in plant-parasitic nematodes. Agricultural fungicides also began as broad-spectrum multi-target compounds, like products containing inorganic sulfur or lime, and have produced limited to no resistance in fungal pathogens^25^. However, as fungicides have developed over time that are targeted against only one biochemical site, like succinate dehydrogenase inhibitors, quinone outside inhibitors, or sterol biosynthesis inhibitors, resistance to these compounds has begun to occur^25^. Sometimes resistance development can lead to products being removed from use^25^. There is no reason to believe that this could not also occur with chemical controls used to manage plant-parasitic nematodes.

This research aimed to provide a greater understanding of how nematicides alter nematode biochemical pathways in *M. incognita* at a transcriptional level. Comprehension of the biological responses that occur due to nematicide exposure is important not only for understanding the functionality of these new nematicides, but could also provide insight into how nematodes might be able to overcome nematicide toxicity in the future. The study used previously generated data (Wram et al. in-press) to explore how oxamyl, fluopyram, fluensulfone, and fluazaindolizine impact biochemical pathways involved in essential metabolic and neuron functionality. The first objective of this study was to understand how each of these nematicides impact the citric acid cycle and oxidative phosphorylation, as these contain the target of fluopyram, a known SDHI inhibitor. The second objective was to explore the impacts of oxamyl, fluopyram, fluensulfone, and fluazaindolizine on fatty acid biosynthesis, which generates critical components for the citric acid cycle. The final objective was to examine the impacts of these compounds on enzymes involved in acetylcholine neuron functionality, the target of oxamyl.

## Results

### $\upbeta$-Fatty acid oxidation

**Figure 1:**
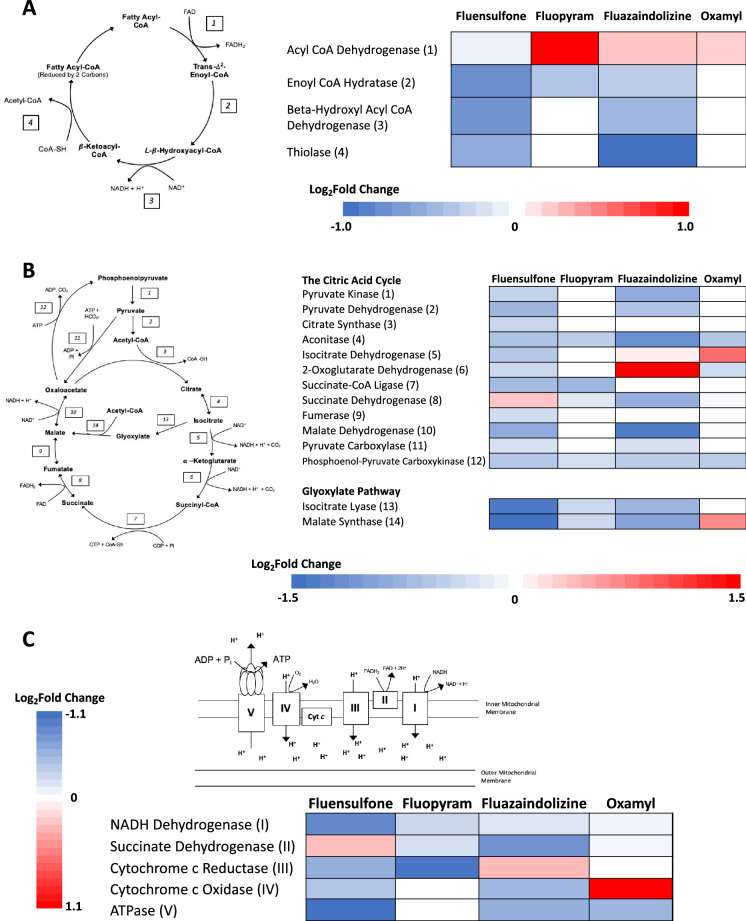
Gene expression summaries of major biochemical pathways in *Meloidogyne incognita* after exposure to nematicides. *Meloidogyne incognita* second-stage juveniles were exposed to fluensulfone, fluopyram, fluazaindolizine, and oxamyl for 24-h and high throughput sequencing used to determine gene expression compared to a water treated control (N = 4 replicates per treatment). In each panel, expression is in the form of Log_2_ Fold Change (Log_2_FC); red and blue indicate upregulated or downregulated expression compared to control, respectively. Each Log_2_FC value shown represents the arithmetic mean of expression of significantly (*P* < 0.05) differently expressed genes that were identified as that enzyme in *M. incognita*. The enzymatic steps in $\upbeta$-fatty acid oxidation are outlined along with their corresponding expressions of each enzyme in the pathway after nematicide exposure in panel (**A**). In panel (**B**), the citric acid cycle and the glyoxylate pathway are outlined with the corresponding expressions of each enzyme in the pathway after nematicide exposure. Panel (**C**), outlines the steps of oxidative phosphorylation and the corresponding expressions of each enzyme in oxidative phosphorylation after nematicide exposure.

$\upbeta$-Fatty acid oxidation is an important process that takes stored fatty acids and transforms them to useful metabolic energy. The output of $\upbeta$-fatty acid oxidation are co-factors for ATP generation (NADH and FADH_2_) and acetyl CoA, which can feed into the TCA cycle, producing more ATP for the cell. The breakdown of fatty acids via $\upbeta$-fatty acid oxidation is completed in four steps outlined in Fig. 1A^26^. Expression of enzymes at each step of $\upbeta$-fatty acid oxidation were, on average, down regulated in *M. incognita* treated with fluensulfone; downregulation of each step ranged from 0.57 to 0.94-fold (Fig. 1A). Unlike *M. incognita* treated with fluensulfone, expression of the first enzyme in the $\upbeta$-fatty acid oxidation cycle, acyl CoA dehydrogenase, was upregulated in *M. incognita* treated with fluopyram, fluazaindolizine, and oxamyl 1.50-, 1.09-, and 1.08-fold, respectively (Fig. 1A). Fluazaindolizine was the only other nematicide, besides oxamyl, that impacted expression of enzymes at each step of in the $\upbeta$-fatty acid oxidation cycle (Fig. 1A). Expression of enzymes used in steps 2–4 were downregulated between 0.49- and 0.78-fold in *M. incognita* treated with fluazaindolizine (Fig. 1A). Individual genes and their expression patterns are shown in Supplemental Fig. 1. Fluensulfone and fluazaindolizine generated the most common differentially expressed genes across the $\upbeta$-fatty acid oxidation cycle, with 16 of the 39 genes differentially expressed by both nematicides.

### The citric acid cycle

The citric acid cycle (TCA) is one of the most critical biochemical pathways in the cell, producing two important reducing equivalents, NADH and FADH_2_, which are used to transfer electrons to the electron transport chain (ETC) and generate ATP through oxidative phosphorylation^27^. Each step of the TCA cycle is outlined in Fig. 1B with the catalyzing enzymes and byproducts produced denoted. All of the nematicides caused differential expression in *M. incognita* of enzymes involved in the TCA cycle. Individual genes and their expression patterns are shown in Supplemental Fig. 2. Fluazaindolizine and fluensulfone had the most considerable impact on the expression of the 12 enzymes in the TCA cycle, with 10 and 12 of the enzymes being differentially expressed, respectively (Fig. 1B). Oxamyl and fluopyram impacted expression of 6 and 4 of the enzymes, respectively (Fig. 1B). In fluensulfone treated *M. incognita* expression of enzymes at each step of the TCA cycle were on average down regulated between 0.53–0.80-fold with the exception of expression of succinate dehydrogenase which was upregulated 1.2-fold (Fig. 1B). Exposure of *M. incognita* to fluazaindolizine resulted in down regulation of expression of 7 of the 12 enzymes involved in the TCA cycle (Fig. 1B). There was on average a slight increase in expression of both isocitrate dehydrogenase and succinate-CoA ligase in *M. incognita* treated with fluazaindolizine, but a greater than a twofold increase in expression of 2-Oxoglutarate dehydrogenase (Fig. 1B). This overexpression of 2-Oxoglutarate dehydrogenase in fluazaindolizine treated *M. incognita* was the largest change in gene expression in all 12 enzymes of the TCA cycle for any of the nematicides. Of the six enzymes in the TCA cycle impacted by oxamyl, all but one was downregulated. The largest change in expression of oxamyl treated *M. incognita* was seen in isocitrate dehydrogenase which was upregulated 1.5-fold (Fig. 1B). Fluopyram induced the least number of changes in expression of genes involved in the TCA cycle, only 4 of the 12 enzymes involved were differentially expressed (Fig. 1B). Aconitase (step 4), succinate-CoA ligase (7), succinate dehydrogenase (8), and phosphoenol-pyruvate carboxykinase (step 12), were downregulated in fluopyram treated *M. incognita,* by 0.69, 0.58, 0.83, and 0.78-fold, respectively (Fig. 1B).

### The glyoxylate pathway

Nematodes can bypass isocitrate dehydrogenase and 2-oxoglutarate dehydrogenase steps in the TCA cycle using the glyoxylate pathway^4,28^. In *M. incognita* treated with fluensulfone, fluazaindolizine, and fluopyram both enzymes in this pathway were downregulated (Fig. 1B). The greatest reduction in expression occurred in *M. incognita* treated with fluensulfone with expression reduced 0.36- and 0.34-fold for isocitrate lyase and malate synthase, respectively. The expression of both isocitrate lyase and malate synthase was reduced 0.75-fold in fluopyram treated *M. incognita*. Expression of isocitrate lyase and malate synthase was reduced by 0.57- and 0.50-fold, respectively, in *M. incognita* treated with fluazaindolizine. The expression of isocitrate lyase in *M. incognita* was unaffected by oxamyl, however, this nematicide did cause the upregulation of malate synthase by 1.4-fold.

### Oxidative phosphorylation

Electrons from the energy-rich molecules generated during the TCA cycle, glycolysis, and fatty-acid oxidation (NADH and FADH_2_) are transferred to O_2_ to generate ATP in a process called oxidative phosphorylation^29^. The steps and resulting products of each step of oxidative phosphorylation are outlined in Fig. 1C. Fluensulfone resulted in the downregulation of NADH dehydrogenase, cytochrome *c* reductase, cytochrome *c* oxidase, and ATPase in *M. incognita* with average expression reduced between 0.45- and 0.72-fold (Fig. 1C). Fluopyram only caused the reduction in expression of the first three enzymes in oxidative phosphorylation, with downregulation averaging between 0.47- and 0.84-fold (Fig. 1C). Both fluazaindolizine and oxamyl resulted in the upregulation of at least one enzyme cytochrome *c* reductase and cytochrome *c* oxidase, respectively. In *M. incognita* treated with fluazaindolizine, cytochrome *c* reductase was upregulated an average of 1.2-fold, with all other enzymes having reduced expression between 0.55- and 0.87-fold (Fig. 1C). The major expression changes resulting from oxamyl were an upregulation of cytochrome *c* oxidase by an average of 2.0-fold and the reduction of ATPase by an average of 0.66-fold (Fig. 1C). Individual genes and their expression patterns are shown in Supplemental Fig. 3. Succinate dehydrogenase, *Minc3s06909g40472*, was the only gene differentially expressed across all nematicides in this pathway (Supplemental Fig. 3). Fluazaindolizine and fluensulfone shared the most common differentially expressed genes, 17 of the 61 genes, and directionality of expression was the same in 14 of the shared differentially expressed genes (Supplemental Fig. 3).

### Acetylcholine neurons

**Figure 2:**
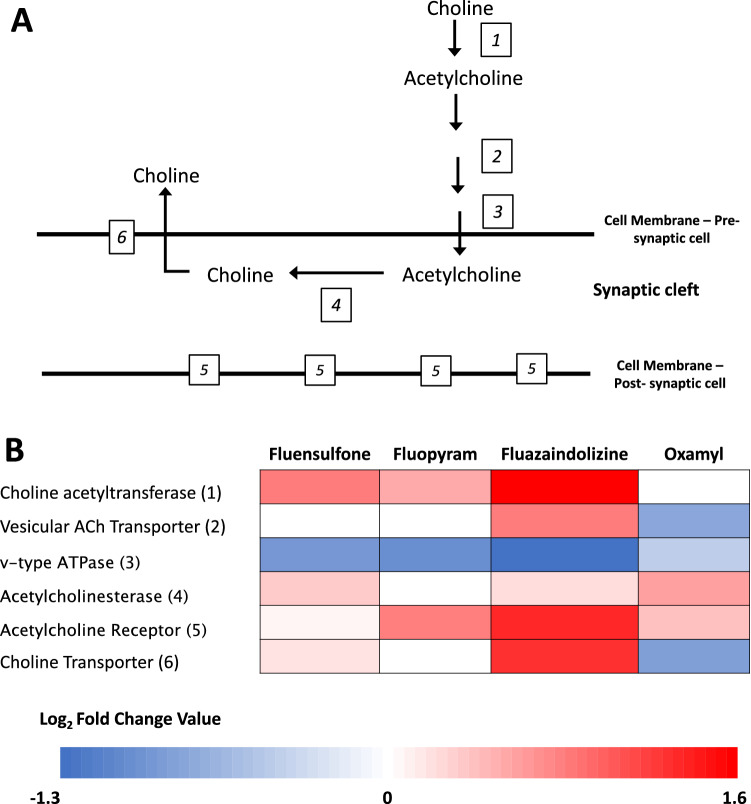
Gene expression summary of acetylcholine neuron components in *Meloidogyne incognita* after nematicide exposure. *Meloidogyne incognita* second-stage juveniles were exposed to fluensulfone, fluopyram, fluazaindolizine, and oxamyl for 24-h and high throughput sequencing used to determine gene expression compared to a water treated control (N = 4 replicates per treatment). Expression is in the form of Log_2_ Fold Change (Log_2_FC); red and blue indicate upregulated and downregulated expression compared to control, respectively. Each Log_2_FC value represents the arithmetic mean of expression of significantly (*P* < 0.05) differently expressed genes that were identified as that enzyme in *M. incognita*. The enzymatic steps involved in acetylcholine neuron potential are outlined in panel (**A**), with the corresponding expression to each step in panel (**B**).

The neurotransmitter acetylcholine is a major component of nematode neuromuscular systems and responsible for many nematode behaviors including movement and feeding^4,30^. Each step of neuron activation and the corresponding enzymes are outlined in Fig. 2A.

On average choline acetyltransferases were upregulated between 1.4 and 3.2-fold in fluopyram, fluensulfone, and fluazaindolizine treated *M. incognita* (Fig. 2B). In oxamyl treated *M. incognita*, choline acetyltransferases were not significantly affected (Fig. 2B). Only fluazaindolizine and oxamyl changed the expression of vesicular acetylcholine transporters in *M. incognita*, with expression increased by 1.81-fold and reduced 0.56-fold, respectively (Fig. 2B). Expression of v-type ATPases was on average downregulated by all of the nematicides between 0.43- and 0.73-fold, with the strongest reduction by fluazaindolizine (Fig. 2B). Acetylcholinesterase and acetylcholine receptors were upregulated in fluensulfone, fluazaindolizine, and oxamyl treated *M. incognita* with only acetylcholine receptors upregulated 1.78-fold by fluopyram (Fig. 2B). Expression of acetylcholinesterase and acetylcholine receptors were on average up-regulated > 1.3-fold in oxamyl treated *M. incognita*. In fluazaindolizine treated *M. incognita*, expression of acetylcholinesterase and acetylcholine receptors were upregulated 1.2 and 2.6-fold (Fig. 2B). Fluensulfone resulted in the 1.2-fold increase in expression of acetylcholinesterase and only 1.05-fold increase in expression of acetylcholine receptors in *M. incognita*. Finally, choline transferases were upregulated 1.1 and 2.5-fold in fluensulfone and fluazaindolizine treated *M. incognita,* respectively*,* but expression was not impacted by fluopyram. The average expression of choline transporters in *M. incognita* treated with oxamyl was downregulated by 0.54-fold (Fig. 2B). Individual genes and their expression patterns are shown in Supplemental Fig. 4.

## Discussion

This is the first study to characterize transcriptomic changes in plant-parasitic nematodes in response to the new nematicides fluopyram, fluensulfone, fluazaindolizine. Using next-generation sequencing we were able to detect changes in gene expression in *M. incognita* that correspond to the known modes-of-action of fluopyram and oxamyl, in addition to the off-target effects of these compounds. We were also able to provide potential targets for the modes-of-action for fluensulfone and fluazaindolizine. Specific expression profiles for the biochemical pathways for β-fatty acid oxidation, the citric acid cycle, oxidative phosphorylation, and acetylcholine neurons and discussed in detail below.

### $\upbeta$-Fatty acid oxidation

We observed that fluensulfone and fluazaindolizine had the greatest impacts on gene expression of enzymes in $\beta$-fatty acid oxidation. This is unsurprising given that fluensulfone and fluazaindolizine shared the most differentially expressed genes of all the nematicides in this study, 488 genes, 10 × greater than any other pairwise comparison of differentially expressed genes (Wram et al. in-press). Fluensulfone and fluazaindolizine both altered the expression of each step in the $\upbeta$-fatty acid oxidation pathway, whereas oxamyl and fluopyram had limited impacts on this pathway.

Kearn et al.^31^ observed that after treating *Globodera pallida* J2 with 10 μM fluensulfone for 10 days there was 50% higher lipid content compared to *G. pallida* J2 that were either untreated or treated with 30 μM aldicarb. Aldicarb in this study served as a comparison of how lipids might accumulate due to nematode paralysis and therefore limited energy expenditure. However, it was also observed that the accumulation of lipids in fluensulfone-treated *G. pallida* J2 surpassed that expected of paralyzed nematodes, suggesting that lipid metabolism was being inhibited by fluensulfone^31^. Although fatty acid content was not measured in this study, synthesis of all enzymes that breakdown fatty acids was significantly reduced by treatment with fluensulfone in *M. incognita* which would reduce the capacity of the nematode to metabolize fatty acids. This corroborates the observed lipid accumulation in *G. pallida*^31^. Further investigation is warranted into examining lipid accumulation after treatment with fluensulfone in *M. incognita,* as well as exploring the sensitivity of *M. incognita* to fluensulfone after knocking out enzymatic expression of each step in this pathway.

### The citric acid cycle

Of the four nematicides examined in this study, fluopyram and oxamyl both have defined modes-of-action. In fungi, fluopyram, is a known inhibitor of succinate dehydrogenase, a key enzyme that plays roles both the TCA cycle and oxidative phosphorylation^32^. Heiken^12^ provided evidence to support the same mode-of-action in nematodes. *Caenorhabditis elegans* succinate dehydrogenase knockdown (*sdh1*) strain VC294 and wild-type strain N2 were exposed to a range of fluopyram concentrations (0.1–10 ppm) for 24 h to generate dose–response curves^12^. Compared to the wild-type *C. elegans*, the EC_50_ (effective dose to kill 50% of exposed population) was reduced from 11.4 to 4.31 ppm in the mutant *C. elegans* VC294 strain, indicating the *sdh1* knockdown *C. elegans* strain VC294 was overly sensitive to fluopyram.

The fungus *Sclerotinia sclerotiorum* was exposed to a novel thiazole carboxamide, a succinate dehydrogenase inhibitor, to examine changes in gene expression after exposure^33^. Aconitase, isocitrate dehydrogenase, 2-oxoglutarate dehydrogenase, succinate-CoA ligase, fumarate hydratase, and malate dehydrogenase were all down regulated along with succinate dehydrogenase subunits A and B with fold changes < 0.67. We also observed similar levels of average downregulation of aconitase, succinate-CoA ligase, and succinate dehydrogenase when *M. incognita* was exposed to fluopyram with fold changes between 0.58 and 0.87. In *S. sclerotiorum*, there was a > 4-fold increase in expression of phosphoenolpyruvate carboxykinase^33^, which was significantly downregulated in this study by fluopyram. Other components of the TCA cycle were not altered in their expression when *M. incognita* was exposed to fluopyram.

Although oxamyl has a defined mode-of-action as an acetylcholinesterase inhibitor, off target effects in other cellular pathways can occur^9^. However, we observed limited impacts on the TCA cycle; within the cycle there were only low levels of downregulation of 5 of the 12 enzymes in the regular TCA cycle. In the fungus *Trichoderma asperellum*, the organophosphate dichlorvos also upregulated isocitrate dehydrogenase after 24 h of exposure^34^. Leung and Meyer^35^ examined 51 different organophosphates and carbamates for their potential binding affinities to mammalian acetylcholinesterases, G-protein coupled receptors, nuclear receptors, cytochrome P450s, along with chemical structure similarities to TCA cycle intermediates. They found that of the 107 receptors they tested, oxamyl only bound to acetylcholinesterases and had low structure similarity to citric acid cycle intermediates, but high structure similarity to acetylcholine. This supports the low impact of oxamyl on the TCA cycle as observed in this study.

Acetyl-CoA, which is generated by the β-fatty acid oxidation pathway, is an allosteric regulator of pyruvate dehydrogenase, wherein, binding of acetyl-CoA activates pyruvate dehydrogenase^27,36^. Low levels of acetyl-CoA that would occur from a reduction in $\upbeta$-fatty acid oxidation activity would decrease the activity of the TCA cycle by reducing the activation of pyruvate dehydrogenase. This could be the phenomenon observed in this study; after treatment of *M. incognita* with fluensulfone, down regulation was observed of almost every enzyme in the TCA cycle, excluding succinate dehydrogenase which was slightly upregulated by 1.1-fold.

Similar to fluensulfone, fluazaindolizine caused the downregulation of many enzymes in the TCA cycle (Fig. 1B). However, 2-oxoglutarate dehydrogenase (alpha-ketoglutarate dehydrogenase) was 2.2-fold upregulated in *M. incognita* treated with fluazaindolizine. This was the highest upregulation of any enzyme in this pathway by any nematicide. Defects in 2-oxoglutarate dehydrogenase functionality are common in a number of neurological diseases in humans including Alzheimer's disease^37^. Decreased activity of 2-oxoglutarate dehydrogenase was found in areas of the brain where neurons died, most likely due to the reactive oxygen species that are produced by 2-oxoglutarate dehydrogenase. Thoden and Wiles^23^ found that *M. incognita* exposed to 50 ppm fluazaindolizine experienced similar body movement symptomology like coiling, z-shape, and j-shape to nematodes exposed to carbamates and organophosphates, whose modes-of-action impact neuron functionality. The impacts of 2-oxoglutarate dehydrogenase inhibition could manifest in pour neuron functionality or death which may be responsible for the odd movement patterns observed in nematodes exposed to fluazaindolizine. There was downregulation/no change of expression of enzymes involved in the steps leading up to 2-oxoglutarate dehydrogenase and then the same downregulation/no change after the 2-oxoglutarate dehydrogenase step in *M. incognita* treated with fluazaindolizine. In *M. incognita* treated with oxamyl, we observed a similar pattern of expression around the target of oxamyl, acetylcholinesterase. *Meloidogyne incognita* treated with fluazaindolizine may be overexpressing 2-oxoglutarate dehydrogenase to compensate for inhibition of this enzyme by fluazaindolizine. This expression pattern provides evidence to support examining 2-oxoglutarate dehydrogenase as a potential mode-of-action for fluazaindolizine.

### The glyoxylate pathway

The glyoxylate pathway provides a way to bypass steps 5 thru 9 in the TCA cycle (Fig. 1B). In fluopyram, fluazaindolizine, and fluensulfone treated *M. incognita*, expression of both isocitrate lyase and malate synthase were downregulated, with the most significant downregulation caused by fluensulfone. Although the glyoxylate pathway plays a role in the TCA cycle, it is also important for carbohydrate anabolism via gluconeogenesis from fatty acids using acetyl-CoA generated from $\upbeta$-fatty acid oxidation^4^. In *Candida albicans,* a yeast species, expression of enzymes in both $\beta$-fatty acid oxidation and glyoxylate pathways were shown to be regulated in the same directionality in response to a variety of differing microenvironments, including in the presence of macrophages^38^. As $\beta$-fatty acid oxidation was decreased in fluensulfone and fluazaindolizine treated *M. incognita*, this coupled regulation could explain the down regulation seen by these nematicides in the glyoxylate pathway. In oxamyl treated *M. incognita*, $\upbeta$-fatty acid oxidation remained mostly unchanged from the untreated control except for the slight average upregulation of acyl CoA dehydrogenase. However, there was also upregulation of malate synthase by 1.4-fold by oxamyl, and no change in isocitrate lyase of the glyoxylate pathway. In *C. elegans*, isocitrate lyase and malate synthase expression were induced in second-stage larvae when nematodes were without food for 36 h^39^. This could contribute to the expression pattern observed in *M. incognita* treated with oxamyl.

### Oxidative phosphorylation

Each nematicide evaluated in this study had an impact on the expression of at least three complexes of oxidative phosphorylation (Fig. 1C). Fluopyram resulted in the downregulation between 0.47- and 0.83-fold of the first three complexes in *M. incognita*, including succinate dehydrogenase, the target of this compound. Similar levels of downregulation were observed in zebra fish treated with thifluzamide, a succinate dehydrogenase inhibitor; downregulation of complexes I–V was between 20–60% at two different doses of thifluzamide^40^. Oxamyl resulted in the average downregulation of complex I, complex II, and complex V in *M. incognita*, a trend that was also observed in *C. elegans* treated with dichlorvos, an acetylcholinesterase inhibitor^41^. In this study complex IV, cytochrome *c* oxidase, was upregulated in *M. incognita* treated with oxamyl. The complex IV reaction is rate limiting for ATP production in oxidative phosphorylation^42^. Additionally, organophosphates have been shown to have inhibitory effects on complex IV activity^43^. In this study, the increased expression of this enzyme in *M. incognita* treated with oxamyl may be an attempt to rescue complex IV activity and ATP production during oxidative phosphorylation.

Fluensulfone and fluazaindolizine downregulated expression of complexes I, IV, and V in *M. incognita*, but upregulated expression of complex II and complex III. *Meloidogyne incognita* treated with fluensulfone and fluazaindolizine may be trying to rescue malfunctioning oxidative phosphorylation by upregulating the expression of these critical components. Additional research is needed to determine if complexes II and III are potential targets for fluensulfone and fluazaindolizine.

### Acetylcholine neurons

Oxamyl is a known acetylcholinesterase inhibitor, binding acetylcholinesterase and preventing the hydrolysis of acetylcholine. In this study, vesicular acetylcholine (ACh) transporters and v-type ATPases were down regulated by 0.56- and 0.73-fold, respectively, in *M. incognita* treated with oxamyl. Acetylcholinesterases and acetylcholine receptors were upregulated 1.5- and 1.3-fold, respectively, and choline transporters were downregulated by 0.54-fold in *M. incognita* treated with oxamyl. Upregulation of acetylcholinesterases in response to organophosphate and carbamate exposure has also been observed in *C. elegans.* Acetylcholinesterase were upregulated in *C. elegans* in response to exposure to the organophosphate phoxim and the carbamate carbaryl between 6 and > 50-fold, respectively^44^. In other organisms, upregulation of acetylcholinesterase was also observed after organophosphate exposure. In the silkworm *Bombyx mori,* acetylcholinesterases were upregulated threefold after 24 h of exposure to phoxim^45^. The upregulation of synthesis of acetylcholine receptors observed in *M. incognita* treated with oxamyl could be due to the increased amount of acetylcholine present in the synaptic-cleft due to acetylcholinesterase inhibition. The inhibition of acetylcholinesterases would also lead to considerably less choline required to be transported back into the pre-synaptic cell and this could explain the downregulation of choline transporter synthesis in oxamyl treated *M. incognita*.

Fluopyram caused the upregulation of the synthesis of choline acetyltransferases and acetylcholine receptors and downregulation of v-type ATPases in *M. incognita*. Fluopyram has shown to cause strong reversible paralytic effects on *M. incognita* after 24 h of exposure^13,14^, therefore, it is not surprising that this compound also alters expression of genes involved in locomotion. Exploration into the impacts of fluopyram beyond its mode-of-action may help to improve our understanding of all the ways fluopyram is able to inhibit nematode reproduction.

Fluensulfone upregulated the expression of choline acetyltransferase, acetylcholinesterase, acetylcholine receptors, and choline transporters in *M. incognita*, while v-type ATPases were downregulated. Fluensulfone has been shown to inhibit movement of *C. elegans* second and third stage larvae (L2 and L3) at a similar rate as aldicarb, a carbamate and acetylcholinesterase inhibitor^19^. Kearn et al.^19^ exposed *unc-17* (vesicular ACh transporter) knockout of *C. elegans* and wild type (WT) *C. elegans* to fluensulfone and aldicarb, a carbamate and acetylcholinesterase inhibitor, for 24 h to observe percentage paralysis. There was no difference in the number of paralyzed nematodes between knockout *unc-17* strain and WT *C. elegans* after exposure to fluensulfone, however, after exposure to aldicarb paralysis was reduced 80% in the knockout *unc-17* strain. In the same study, fluensulfone also inhibited movement of *C. elegans* L2/L3. We found no change in the expression of vesicular ACh transporters in *M. incognita* after exposure to fluensulfone compared to the control. However, the impacts of fluensulfone on the expression of other components of acetylcholine neuron and the similarity in the physiological impacts between fluensulfone and aldicarb on *C. elegans* provide support for investigating further the impact of fluensulfone on *M. incognita* acetylcholine neurons.

Lahm et al.^22^ used in vitro assays to determine if fluazaindolizine had a similar mode-of-action to current nematicides and found that fluazaindolizine did not affect acetylcholinesterase, mitochondrial electron transport chain, nicotinic acetylcholine receptors, or glutamate-gated chloride channels. As previously described, Thoden and Wiles^23^ observed strong effects of fluazaindolizine on *M. incognita* body movements. Impacts on nematode movement are supported by our findings where fluazaindolizine had the strongest impacts on expression of all components of acetylcholine neurons in *M. incognita*. Synthesis of choline acetyltransferases, vesicular ACh transporters, acetylcholinesterase, acetylcholine receptors, and choline transporters were upregulated between 1.2 and 3.2-fold compared to the water control in *M. incognita* treated with fluazaindolizine. Similar to all other nematicides used in this study, v-type ATPases were also downregulated in by fluazaindolizine. Further research is needed to understand if fluazaindolizine is potentially targeting acetylcholine neurons as a possible mode-of-action or if this is a side-effect of the true mode-of-action of this nematicide.

## Conclusion

In this study *M. incognita* J2 were exposed to fluensulfone, fluazaindolizine, fluopyram, and oxamyl for 24 h to provide a greater understanding of how these nematicides are toxic to nematodes through observing their impacts on gene expression of major biochemical pathways. Gene expression patterns consistent with the known modes-of-action of both fluopyram and oxamyl, and expression changes that were a result of the downstream effects of these modes-of-actions were observed. We also found that fluensulfone and fluazaindolizine had the greatest impact on gene expression of all the biochemical pathways investigated in this study. The expression patterns observed in this study provide opportunities for future research into potential modes-of-action for these compounds; $\upbeta$-fatty acid oxidation pathway and 2-oxoglutarate dehydrogenase of the TCA cycle for fluensulfone and fluazaindolizine, respectively. However, fluazaindolizine may have more than one target given the strong impacts of this nematicide on expression of acetylcholine neuron components. Further research is needed to confirm the mode-of-actions of these important nematicides.

## Materials and methods

The data used in this study was generated for a previous study examining the general trends of transcription of *M. incognita* exposed to fluopyram, fluensulfone, fluazaindolizine, and oxamyl and the impacts of these nematicides on the expression of genes involved in nematode detoxification and fatty-acid retinoid binding (Wram et al. 2021, in-press). Detailed methods can be found in the (Wram et al. 2021, in-press), and are summarized below.

### Nematode collection

To establish *M. incognita* in culture for experimental use nematodes were originally collected from grape (*Vitis vinifera*) in Parlier, CA and placed on a single egg mass on the roots of tomato (*Solanum lycopersicum*) ‘Rutgers’. *Meloidogyne incognita* second-stage juveniles (J2) were obtained by hand-picking egg masses from culture tomato roots and hatched using methods described in^46,47^. Hatched *M. incognita* J2 were collected after 3 days for use in nematicide exposure and RNA sequencing.

### Nematicide exposure

The doses used for nematicide exposure of *M. incognita* varied by compound. For oxamyl, fluopyram, fluensulfone, and fluazaindolizine the exposure doses were 63, 2, 200, and 208 ppm, respectively. *Meloidogyne incognita* J2 (5,000) were exposed in a 1.7 ml microcentrifuge tube with the appropriate concentration of nematicide solution or water for the control, and incubated for 24-h at room temperature. Each nematicide, including the water control, was replicated four times. After 24-h, nematodes were frozen in liquid nitrogen and stored at − 80 °C until RNA was extracted.

### RNA extraction, library preparation, and sequencing

To extract RNA, the RNeasy Minikit^®^ (QIAGEN, Hilden, Germany) was used. Modifications to the kit instructions and more detailed extraction methods can be found in Wram et al. in-press. Oregon State University Center for Genome Research and Biocomputing was used to prep extracted RNA for cDNA libraries using the NEBNext^®^ Ultra RNA Library Prep Kit Illumina (Illumina, San Diego, CA). Before sequencing, RNA was quality checked using a Eukaryote Total RNA Pico chip on a Agilent Bioanalyzer 2100; all samples had an RIN value above 9. Sequencing of the cDNA libraries was performed on the Illumina HiSeq 3000 Platform, using 100 base pair single-end reads.

### Bioinformatic workflow

Illumina adapters and reads with a Phred score < 20 were removed from the sequencing data using Trim Galore!^48^. Remaining reads were aligned to the *M. incognita* genome v3 (NCBI BioProject PRJEB8714)^49^ and reads per gene were counted using STAR^50^. Gene expression and data visualization was done using R^51^ and Microsoft PowerPoint. DESeq2^52^ was used to determine differential gene expression and adjusted p-values with count data output by STAR. Significant differentially expressed genes were genes that had an adjusted p-value < 0.05.

To enable the exploration of expression genes in different biochemical pathways, WormBase ParaSite^53^ was used to download gene identifications that correspond to enzymes in each biochemical pathway (TCA cycle, beta-fatty acid oxidation, oxidative phosphorylation, or cholinergic metabolism) either through their corresponding *C. elegans* ortholog or appropriate pFam domain. To summarize expression across multiple genes with identities to the same enzyme, arithmetic mean was taken of the Log_2_Fold Change of genes with adjusted p-values < 0.05.

Raw sequencing data can be found on NCBI BioProject PRJNA818683.

All local, national, and international guidelines were adhered to in the production of this study. Any plant material that mentioned was used with permission.

## Supplementary Information

Supplementary Figure 1.

Supplementary Figure 2.

Supplementary Figure 3.

Supplementary Figure 4.

Supplementary Information.

Supplementary Legends.
