# Supplementary material for: Transcriptional changes of biochemical pathways in Meloidogyne incognita in response to non-fumigant nematicides
Source: Sci Rep. 2022 Jun 14;12:9875. doi: 10.1038/s41598-022-14091-3 (PMC9197979; doi:10.1038/s41598-022-14091-3)
Supplement: Supplementary file 1 — Supplementary Figure 1. [file 41598_2022_14091_MOESM1_ESM.pdf]

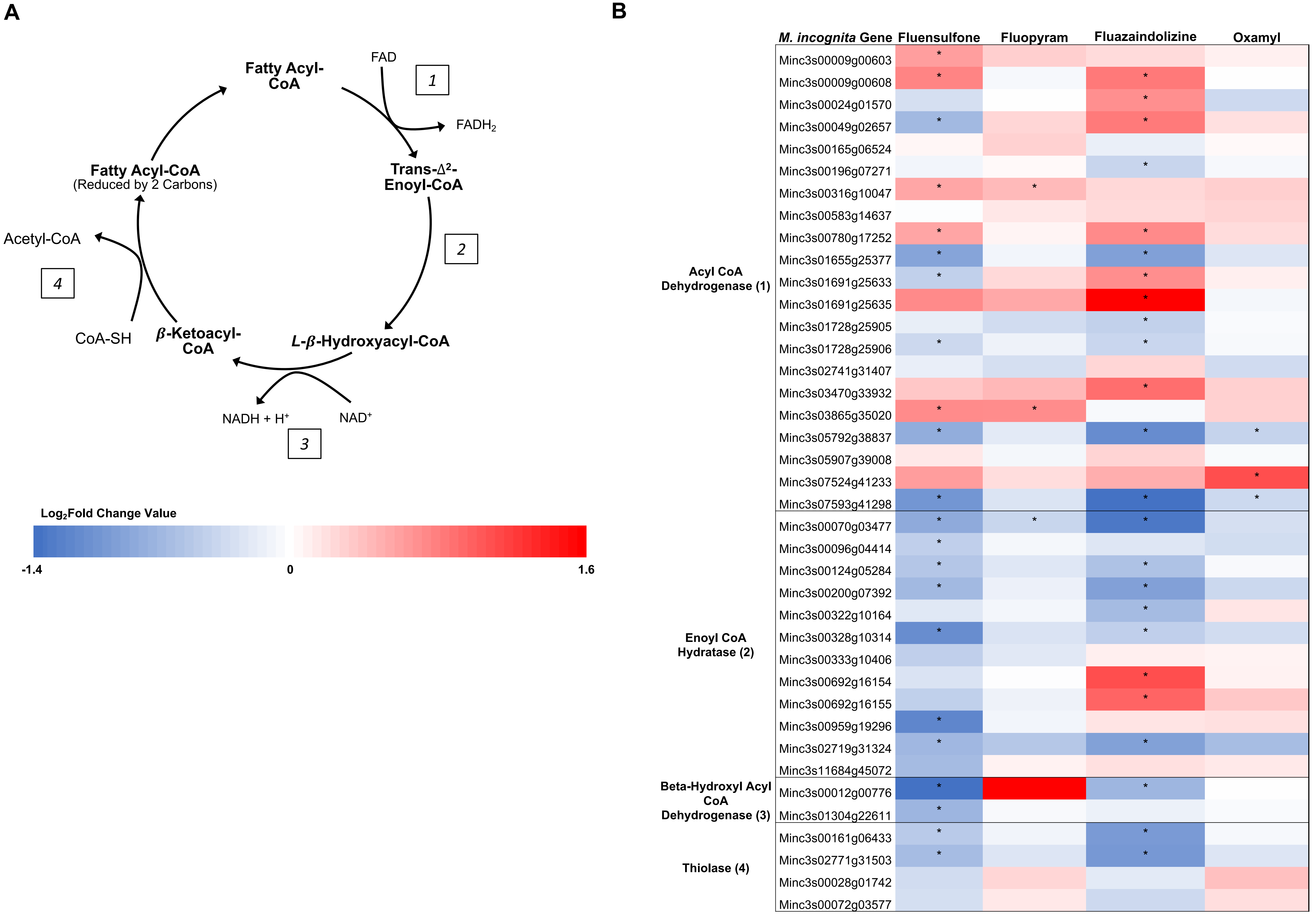
A
Fatty Acyl-CoA
FAD
FADH2
1
Trans-Δ²-Enoyl-CoA
2
L-β-Hydroxyacyl-CoA
NAD+
NADH + H+
3
β-Ketoacyl-CoA
CoA-SH
Acetyl-CoA
4
Fatty Acyl-CoA (Reduced by 2 Carbons)
Log2Fold Change Value
-1.4
0
1.6
B
M. incognita Gene
Fluensulfone
Fluopyram
Fluazaindolizine
Oxamyl
Acyl CoA Dehydrogenase (1)
Minc3s00009g00603
Minc3s00009g00608
Minc3s00024g01570
Minc3s00049g02657
Minc3s00165g06524
Minc3s00196g07271
Minc3s00316g10047
Minc3s00583g14637
Minc3s00780g17252
Minc3s01655g25377
Minc3s01691g25633
Minc3s01691g25635
Minc3s01728g25905
Minc3s01728g25906
Minc3s02741g31407
Minc3s03470g33932
Minc3s03865g35020
Minc3s05792g38837
Minc3s05907g39008
Minc3s07524g41233
Minc3s07593g41298
Enoyl CoA Hydratase (2)
Minc3s00070g03477
Minc3s00096g04414
Minc3s00124g05284
Minc3s00200g07392
Minc3s00322g10164
Minc3s00328g10314
Minc3s00333g10406
Minc3s00692g16154
Minc3s00692g16155
Minc3s00959g19296
Minc3s02719g31324
Minc3s11684g45072
Beta-Hydroxyl Acyl CoA Dehydrogenase (3)
Minc3s00012g00776
Minc3s01304g22611
Thiolase (4)
Minc3s00161g06433
Minc3s02771g31503
Minc3s00028g01742
Minc3s00072g03577
