# Supplementary material for: Transcriptional changes of biochemical pathways in Meloidogyne incognita in response to non-fumigant nematicides
Source: Sci Rep. 2022 Jun 14;12:9875. doi: 10.1038/s41598-022-14091-3 (PMC9197979; doi:10.1038/s41598-022-14091-3)
Supplement: Supplementary file 2 — Supplementary Figure 2. [file 41598_2022_14091_MOESM2_ESM.pdf]

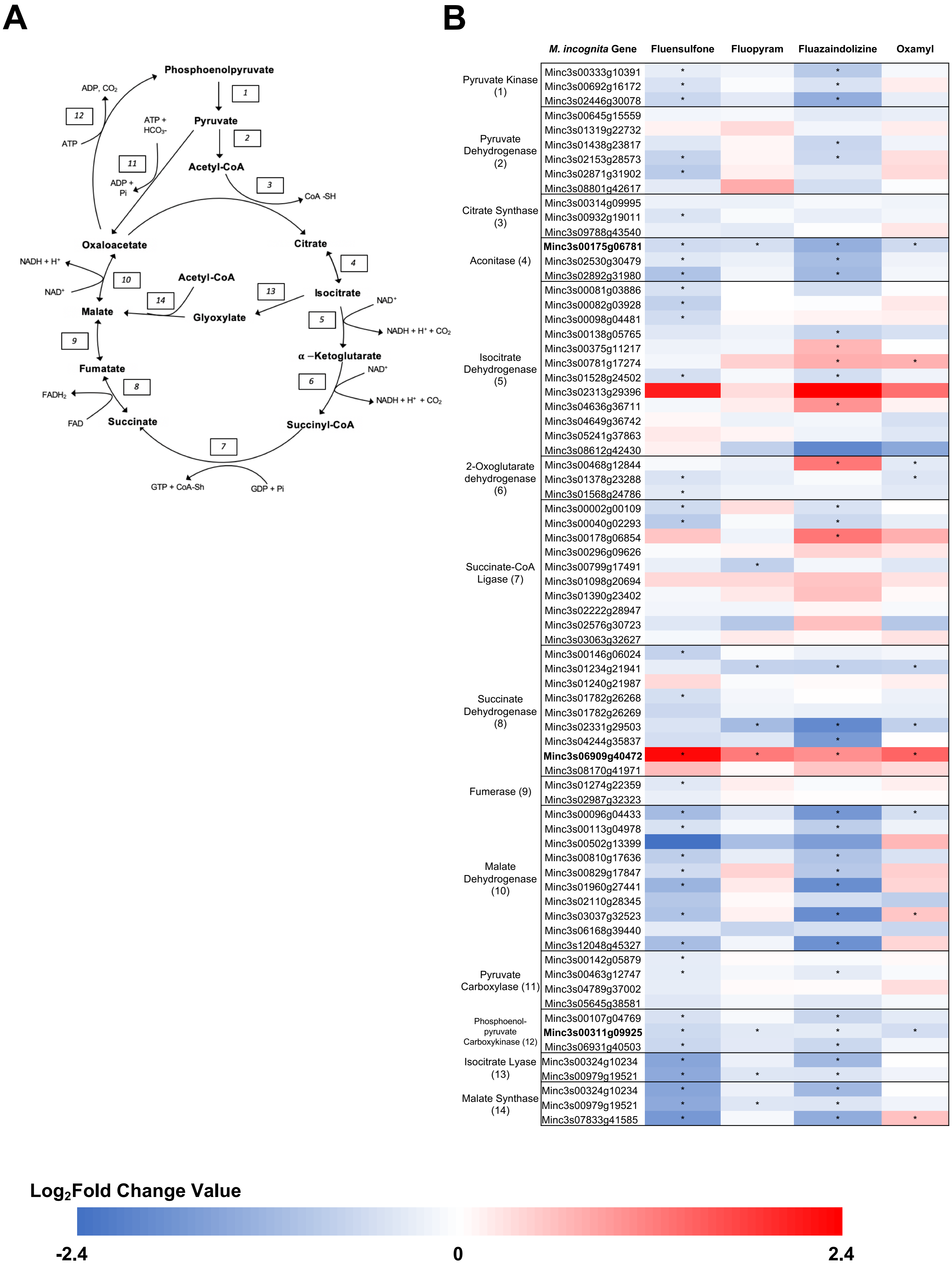
A
Phosphoenolpyruvate
Pyruvate
Acetyl-CoA
CoA -SH
Citrate
Isocitrate
α –Ketoglutarate
Succinyl-CoA
Succinate
Fumatate
Malate
Oxaloacetate
Acetyl-CoA
Glyoxylate
ADP, CO_2
ATP
ATP + HCO_3-
ADP + Pi
NADH + H⁺
NAD⁺
NAD⁺
NADH + H⁺ + CO_2
NAD⁺
NADH + H⁺ + CO_2
GTP + CoA-Sh
GDP + Pi
$FADH_2$
FAD
1
2
3
4
5
6
7
8
9
10
11
12
13
14
B
M. incognita Gene
Fluensulfone
Fluopyram
Fluazaindolizine
Oxamyl
Pyruvate Kinase (1)
Minc3s00333g10391
Minc3s00692g16172
Minc3s02446g30078
Pyruvate Dehydrogenase (2)
Minc3s00645g15559
Minc3s01319g22732
Minc3s01438g23817
Minc3s02153g28573
Minc3s02871g31902
Minc3s08801g42617
Citrate Synthase (3)
Minc3s00314g09995
Minc3s00932g19011
Minc3s09788g43540
Aconitase (4)
Minc3s00175g06781
Minc3s02530g30479
Minc3s02892g31980
Isocitrate Dehydrogenase (5)
Minc3s00081g03886
Minc3s00082g03928
Minc3s00098g04481
Minc3s00138g05765
Minc3s00375g11217
Minc3s00781g17274
Minc3s01528g24502
Minc3s02313g29396
Minc3s04636g36711
Minc3s04649g36742
Minc3s05241g37863
Minc3s08612g42430
2-Oxoglutarate dehydrogenase (6)
Minc3s00468g12844
Minc3s01378g23288
Minc3s01568g24786
Succinate-CoA Ligase (7)
Minc3s00002g00109
Minc3s00040g02293
Minc3s00178g06854
Minc3s00296g09626
Minc3s00799g17491
Minc3s01098g20694
Minc3s01390g23402
Minc3s02222g28947
Minc3s02576g30723
Minc3s03063g32627
Succinate Dehydrogenase (8)
Minc3s00146g06024
Minc3s01234g21941
Minc3s01240g21987
Minc3s01782g26268
Minc3s01782g26269
Minc3s02331g29503
Minc3s04244g35837
Minc3s06909g40472
Minc3s08170g41971
Fumerase (9)
Minc3s01274g22359
Minc3s02987g32323
Malate Dehydrogenase (10)
Minc3s00096g04433
Minc3s00113g04978
Minc3s00502g13399
Minc3s00810g17636
Minc3s00829g17847
Minc3s01960g27441
Minc3s02110g28345
Minc3s03037g32523
Minc3s06168g39440
Minc3s12048g45327
Pyruvate Carboxylase (11)
Minc3s00142g05879
Minc3s00463g12747
Minc3s04789g37002
Minc3s05645g38581
Phosphoenol-pyruvate Carboxykinase (12)
Minc3s00107g04769
Minc3s00311g09925
Minc3s06931g40503
Isocitrate Lyase (13)
Minc3s00324g10234
Minc3s00979g19521
Malate Synthase (14)
Minc3s00324g10234
Minc3s00979g19521
Minc3s07833g41585
Log_2Fold Change Value
-2.4
0
2.4
