# Supplementary figures and images for: Transcriptional changes of biochemical pathways in Meloidogyne incognita in response to non-fumigant nematicides
Source: Sci Rep. 2022 Jun 14;12:9875. doi: 10.1038/s41598-022-14091-3 (PMC9197979; doi:10.1038/s41598-022-14091-3)

A

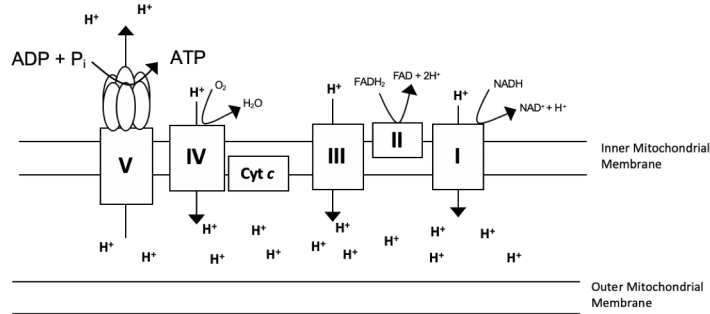

B

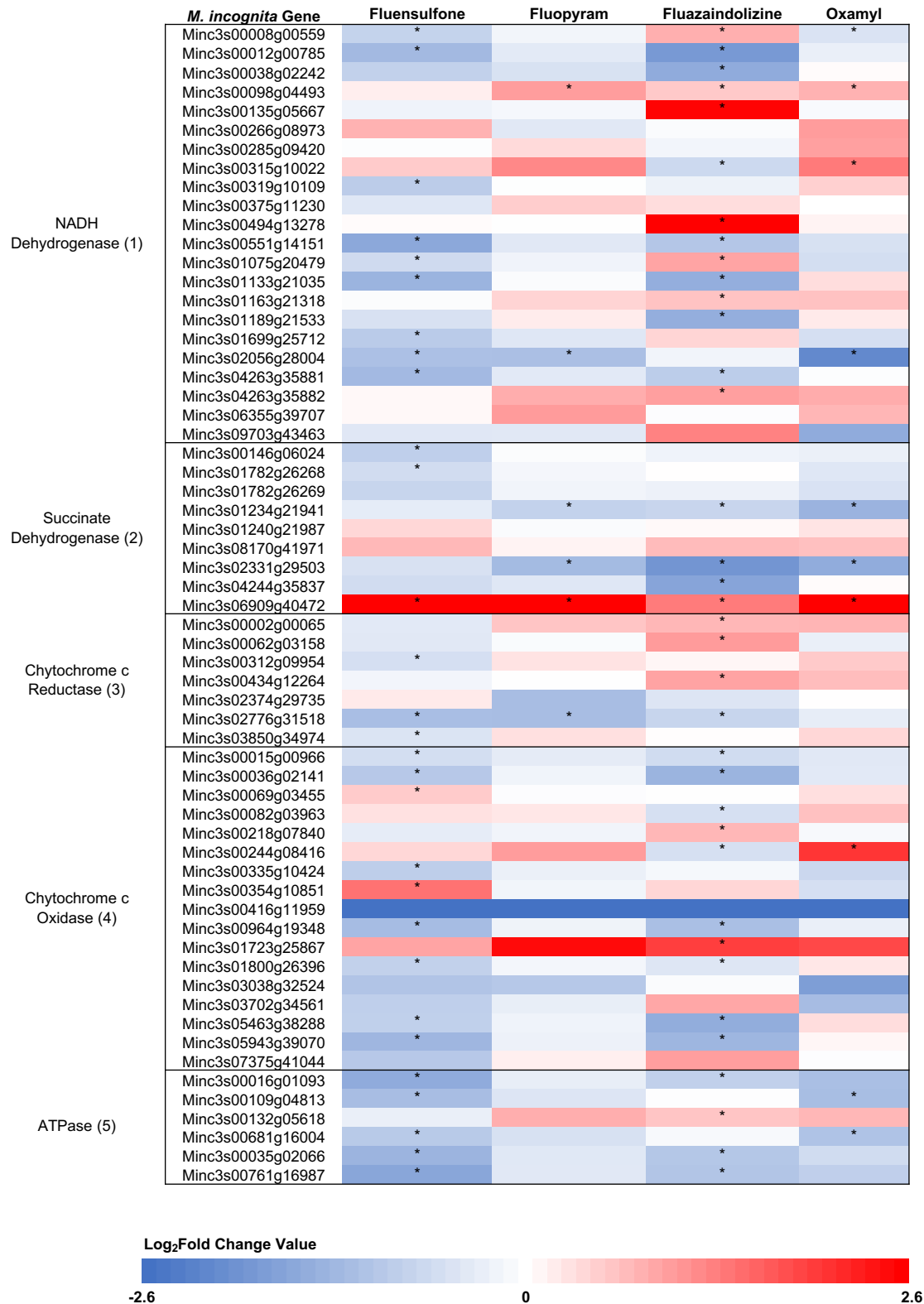

Supplement: Supplementary file 3 — Supplementary Figure 3. [file 41598_2022_14091_MOESM3_ESM.pdf]
