# Supplementary material for: Transcriptional changes of biochemical pathways in Meloidogyne incognita in response to non-fumigant nematicides
Source: Sci Rep. 2022 Jun 14;12:9875. doi: 10.1038/s41598-022-14091-3 (PMC9197979; doi:10.1038/s41598-022-14091-3)
Supplement: Supplementary file 4 — Supplementary Figure 4. [file 41598_2022_14091_MOESM4_ESM.pdf]

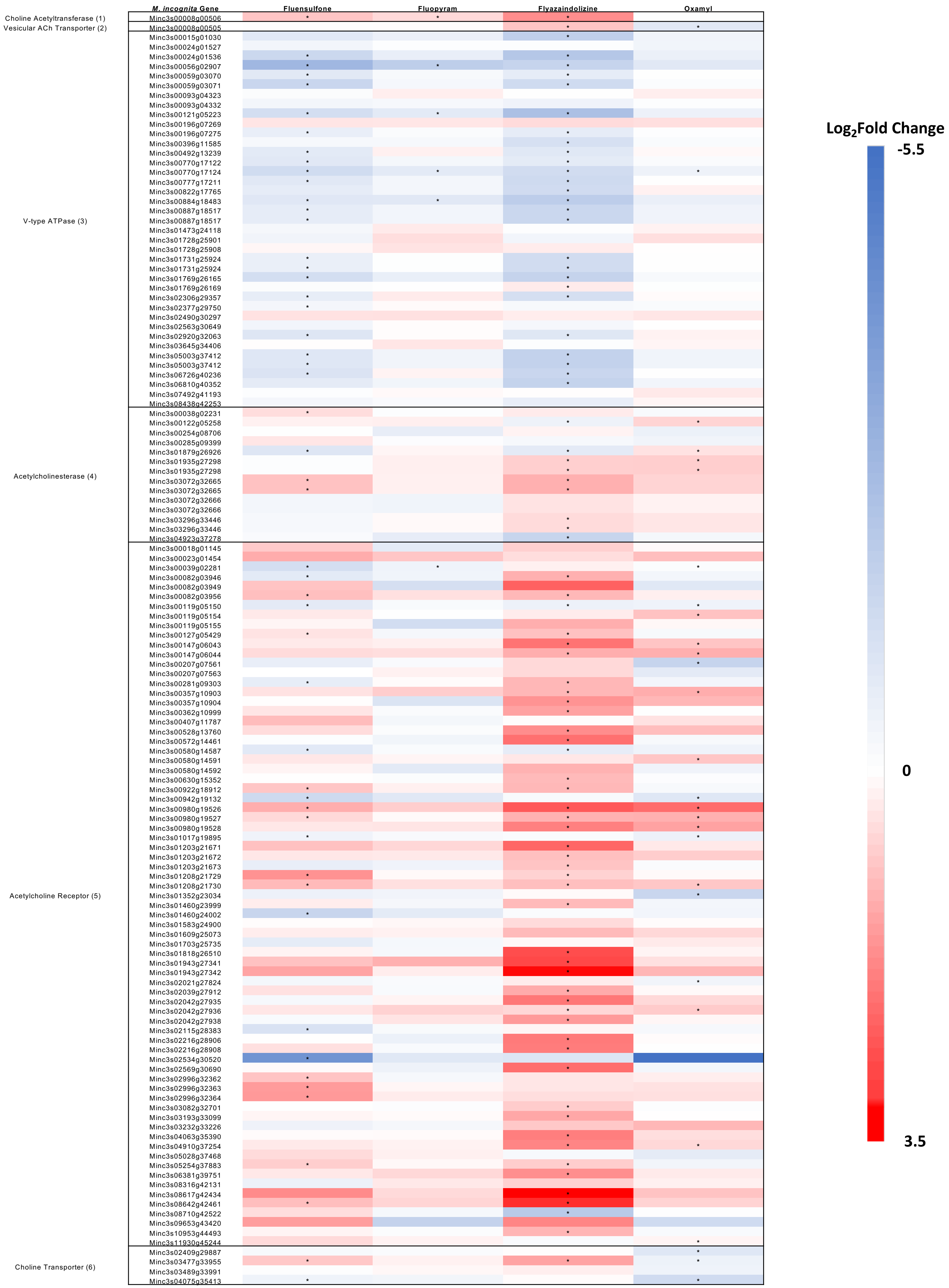
M. incognita Gene
Fluensulfone
Fluopyram
Flyazaindolizine
Oxamyl
Choline Acetyltransferase (1)
Vesicular ACh Transporter (2)
V-type ATPase (3)
Acetylcholinesterase (4)
Acetylcholine Receptor (5)
Choline Transporter (6)
Minc3s00008g00506
Minc3s00008g00505
Minc3s00015g01030
Minc3s00024g01527
Minc3s00024g01536
Minc3s00056g02907
Minc3s00059g03070
Minc3s00059g03071
Minc3s00093g04323
Minc3s00093g04332
Minc3s00121g05223
Minc3s00196g07269
Minc3s00196g07275
Minc3s00396g11585
Minc3s00492g13239
Minc3s00770g17122
Minc3s00770g17124
Minc3s00777g17211
Minc3s00822g17765
Minc3s00884g18483
Minc3s00887g18517
Minc3s00887g18517
Minc3s01473g24118
Minc3s01728g25901
Minc3s01728g25908
Minc3s01731g25924
Minc3s01731g25924
Minc3s01769g26165
Minc3s01769g26169
Minc3s02306g29357
Minc3s02377g29750
Minc3s02490g30297
Minc3s02563g30649
Minc3s02920g32063
Minc3s03645g34406
Minc3s05003g37412
Minc3s05003g37412
Minc3s06726g40236
Minc3s06810g40352
Minc3s07492g41193
Minc3s08438g42253
Minc3s00038g02231
Minc3s00122g05258
Minc3s00254g08706
Minc3s00285g09399
Minc3s01879g26926
Minc3s01935g27298
Minc3s01935g27298
Minc3s03072g32665
Minc3s03072g32665
Minc3s03072g32666
Minc3s03072g32666
Minc3s03296g33446
Minc3s03296g33446
Minc3s04923g37278
Minc3s00018g01145
Minc3s00023g01454
Minc3s00039g02281
Minc3s00082g03946
Minc3s00082g03949
Minc3s00082g03956
Minc3s00119g05150
Minc3s00119g05154
Minc3s00119g05155
Minc3s00127g05429
Minc3s00147g06043
Minc3s00147g06044
Minc3s00207g07561
Minc3s00207g07563
Minc3s00281g09303
Minc3s00357g10903
Minc3s00357g10904
Minc3s00362g10999
Minc3s00407g11787
Minc3s00528g13760
Minc3s00572g14461
Minc3s00580g14587
Minc3s00580g14591
Minc3s00580g14592
Minc3s00630g15352
Minc3s00922g18912
Minc3s00942g19132
Minc3s00980g19526
Minc3s00980g19527
Minc3s00980g19528
Minc3s01017g19895
Minc3s01203g21671
Minc3s01203g21672
Minc3s01203g21673
Minc3s01208g21729
Minc3s01208g21730
Minc3s01352g23034
Minc3s01460g23999
Minc3s01460g24002
Minc3s01583g24900
Minc3s01609g25073
Minc3s01703g25735
Minc3s01818g26510
Minc3s01943g27341
Minc3s01943g27342
Minc3s02021g27824
Minc3s02039g27912
Minc3s02042g27935
Minc3s02042g27936
Minc3s02042g27938
Minc3s02115g28383
Minc3s02216g28906
Minc3s02216g28908
Minc3s02534g30520
Minc3s02569g30690
Minc3s02996g32362
Minc3s02996g32363
Minc3s02996g32364
Minc3s03082g32701
Minc3s03193g33099
Minc3s03232g33226
Minc3s04063g35390
Minc3s04910g37254
Minc3s05028g37468
Minc3s05254g37883
Minc3s06381g39751
Minc3s08316g42131
Minc3s08617g42434
Minc3s08642g42461
Minc3s08710g42522
Minc3s09653g43420
Minc3s10953g44493
Minc3s11930g45244
Minc3s02409g29887
Minc3s03477g33955
Minc3s03489g33991
Minc3s04075g35413
Log2Fold Change
-5.5
0
3.5
