# Supplementary material for: Transcriptional changes of biochemical pathways in Meloidogyne incognita in response to non-fumigant nematicides
Source: Sci Rep. 2022 Jun 14;12:9875. doi: 10.1038/s41598-022-14091-3 (PMC9197979; doi:10.1038/s41598-022-14091-3)
Supplement: Supplementary file 6 — Supplementary Legends. [file 41598_2022_14091_MOESM6_ESM.docx]

**Supplementary Figure 1. Gene expression of** $\boldsymbol{\beta}$**-fatty acid oxidation in *Meloidogyne incognita* after nematicide exposure**. *Meloidogyne incognita* second-stage juveniles (J2) were exposed to fluensulfone, fluopyram, fluazaindolizine, and oxamyl for 24-hrs and high throughput sequencing used to determine gene expression compared to a water treated control (N=4 replicates per treatment). Expression is in the form of Log_2_ Fold Change (Log_2_FC); red indicates upregulated expression compared to control and blue indicates downregulation. Asterisks represent significantly differentially expressed genes (*P* < 0.05). The enzymatic steps involved $\beta$-fatty acid oxidation are outline in panel A with the corresponding expression of each gene identified to a specific enzyme of each step in panel B.

**Supplementary Figure 2. Gene expression of the citric acid cycle and glyoxylate pathway in *Meloidogyne incognita* after nematicide exposure.** *Meloidogyne incognita* second-stage juveniles (J2) were exposed to fluensulfone, fluopyram, fluazaindolizine, and oxamyl for 24-hrs and high throughput sequencing used to determine gene expression compared to a water treated control (N=4 replicates per treatment). Expression is in the form of Log_2_ Fold Change (Log_2_FC); red indicates upregulated expression compared to control, and blue indicates downregulation. Asterisks represent significantly differentially expressed genes (*P* < 0.05). Bolded genes are common significantly differentially expressed genes across all treatments. The enzymatic steps involved in citric acid cycle and glyoxylate are outlined in panel A with the corresponding expression of each gene identified to a specific enzyme of each step in panel B.

**Supplementary Figure 3. Gene expression of oxidative phosphorylation in *Meloidogyne incognita* after nematicide exposure.** *Meloidogyne incognita* second-stage juveniles (J2) were exposed to fluensulfone, fluopyram, fluazaindolizine, and oxamyl for 24-hrs and high throughput sequencing used to determine gene expression compared to a water treated control (N=4 replicates per treatment). Expression is in the form of Log_2_ Fold Change (Log_2_FC); red indicates upregulated expression compared to control and blue indicates downregulation. Asterisks represent significantly differentially expressed genes (*P* < 0.05). The enzymatic steps involved in oxidative phosphorylation are outline in panel A with the corresponding expression of each gene identified to a specific enzyme of each step in panel B.

**Supplementary Figure 4. Gene expression of acetylcholine neuron componets in *Meloidogyne incognita* after nematicide exposure**. *Meloidogyne incognita* second-stage juveniles (J2) were exposed to fluensulfone, fluopyram, fluazaindolizine, and oxamyl for 24-hrs and high throughput sequencing used to determine gene expression compared to a water treated control (N=4 replicates per treatment). Expression is in the form of Log_2_ Fold Change (Log_2_FC); red indicates upregulated expression compared to control and blue indicates downregulation. Asterisks represent significantly differentially expressed genes (*P* < 0.05).
